# Fundamental motor skill interventions significantly improve executive functions and social–emotional competence in preschoolers: a meta-analysis

**DOI:** 10.3389/fpsyg.2025.1721589

**Published:** 2026-01-09

**Authors:** Yuan Li, Hui Yin Ler, Dong Zhang, Lan Su

**Affiliations:** 1 Faculty of Physical Education, Quanzhou Normal University, Quanzhou, China; 2 Faculty of Applied Sciences, Tunku Abdul Rahman University of Management and Technology, Kuala Lumpur, Malaysia; 3 Faculty of Arts and Design, Quanzhou Normal University, Quanzhou, China

**Keywords:** fundamental motor skills, motor skills intervention, executive functions, social-emotional competence, preschool children, early childhood, systematic meta-analysis

## Abstract

Dynamic and embodied cognition theories propose synchronized motor, cognitive, and affective development; however, evidence on whether fundamental motor skills (FMS) enhance executive functions (EFs) and social–emotional competence (SEC) in preschoolers remains inconsistent. This systematic review (PRISMA-guided) synthesized 2000–2025 evidence from 10 studies (n = 2,039; 6 good, 4 fair quality via PEDro) on FMS interventions in typically developing preschoolers. The meta-analysis revealed significant overall EF (SMD = 0.40, 95% CI 0.20–0.61, *p* < 0.001) and SEC (SMD = 0.16, 95% CI 0.03–0.20, *p* = 0.02) improvements. Subgroup analyses suggested that pure FMS programs may yield stronger EF effects (SMD = 0.49) than combined programs (SMD = 0.32), whereas intervention type had a minimal differential impact on SEC outcomes. 5 -year-old benefited more than 3–4-year-old did in both domains (EFs: 0.49 vs. 0.36; SECs: 0.22 vs. 0.12). A higher dosage (>2 sessions/week × 30 min) yielded greater EF gains (0.51 vs. 0.27), while no significant dose–response relationship was observed for SEC. The findings confirm that FMS interventions enhance preschoolers’ EFs and SEC, with optimal EFs from pure FMS targeting older children at higher dosages; SEC benefits are consistent across intervention types.

**Systematic review registration:**

The protocol for this systematic review was prospectively registered on PROSPERO with the Unique Identifier: [CRD420251073707]. The registration is publicly accessible at: https://www.crd.york.ac.uk/prospero/, identifier CRD420251073707.

## 1 Introduction

Human development includes four domains: physical, motor, cognitive and affective. These domains are not discrete; they are in constant interaction with each other (Hulteen et al., 2018; Payne and Isaacs, 2017; Seefeldt and Haubenstricker, 1982). While national policies vary in defining the preschool age range, such as 3–5 years (Australian Government Department of Health, 2019; US. Centers for Disease Control and Prevention, 2024), 3–6 years (Ministry of Education China, 2012a), or 4–6 years (Ministry of Education Malaysia, 2017), the consensus globally encompasses children aged 3–6 years. Proactive interventions during this critical period yield long-term positive effects on developmental trajectories (UNICEF, 2022). Crucially, this period represents a sensitive transitional stage in fundamental motor skill (FMS) development, where children typically progress from the initial to the intermediate phase (Clark and Metcalfe, 2002). During this window, improvements in cognitive and emotional processes are highly responsive to environmental stimulation and structured practice (Piaget and Cook, 1952; Vygotsky and Cole, 1978). Two theoretical paradigms elucidate the interconnections of these developmental domains. Dynamic systems theory (Thelen and Smith, 1994) posits that motors and cognition engage in continuous, bidirectional interactions, mutually shaping their co-emerging developmental pathways. Embodied cognition theory demonstrates how bodily states, sensory systems, and motor actions scaffold higher-order psychological processes—including language comprehension, memory, and social interaction (Glenberg, 2010). These theories posit that motor experiences are fundamental to the development of cognitive, emotional processes. The physical act of learning and controlling movement directly engages and strengthens core executive functions. Simultaneously, navigating the challenges and successes of motor tasks provides vital opportunities to practice emotional regulation through a sense of competence.

Fundamental motor skills (FMS) represent learned movement patterns requiring explicit instruction, forming the foundation for advanced physical activities and sport performance, which consists of locomotor skills (e.g., running), stability skills (e.g., bending), and object-control (OC) skills (e.g., dribbling) (Barnett et al., 2016; Logan et al., 2018). Executive functions (EFs) comprise top-down cognitive processes that regulate goal-directed behaviors (Diamond, 2013). Miyake et al. (2000) proposed that EFs comprise core skills including inhibition (suppressing inappropriate responses), cognitive flexibility (shifting between ideas and activities), and working memory (holding, updating, and actively manipulating information). Social–emotional competence (SEC) encompasses the integrated ability to form meaningful relationships, self-regulate emotional experiences, and interact with learning environments in culturally responsive ways (Yates et al., 2008).

Emerging evidence suggests that fundamental motor skill (FMS) interventions have more substantial effects on preschool children’s cognitive and academic outcomes than does general physical activity (Jylänki et al., 2022). Several studies have demonstrated the beneficial effects of motor skill interventions on cognitive functions such as memory, inhibition, and conversion, as well as academic ability (Capio et al., 2022; Malambo et al., 2022; Martins et al., 2020; Vanhala et al., 2022). Notably, FMS has been shown to be positively correlated with emotion and social skills (Jang and Hong, 2022), especially in body-directed games (Dias Rodrigues et al., 2022). Children with developmental coordination disorder (DCD) demonstrated significantly poorer FMS (Yu et al., 2021). Interventions focusing exclusively on motor skill development are termed “Pure FMS” interventions (Capio et al., 2024; Miller et al., 2023), while those centered on motor development but incorporating strategies to enhance EFs or SEC are referred to as “Combined FMS” interventions (Brian et al., 2024; Cunningham et al., 2025).

Many empirical studies have demonstrated the evidence-supported outcomes of FMS interventions for enhancing children’s executive function and SEC development, although the findings remain inconsistent. While studies by Mulvey et al. (2018) and Miller et al. (2023) reported positive effects of FMS training on executive function, contradictory results emerged in the works of Sendil et al. (2024) and Cunningham et al. (2025). Similarly, Ortin et al. (2024) and Brian et al. (2024) documented improvements in social–emotional competencies following FMS interventions, whereas Vazou and Mavilidi (2021) and Capio et al. (2024) failed to replicate these benefits. Meta-analyses consistently indicate heterogeneous effects of FMS-integrated physical activity interventions across EFs and social–emotional outcomes (Vazou et al., 2019; Xue et al., 2019). This variability may stem from multiple moderating factors, including quality of movement demonstration, adequacy of practice time, and progressive challenge design in task complexity (Diamond and Ling, 2016). Critical knowledge gaps persist regarding dose–response relationships (Li and Xiang, 2023), and the efficacy of combined FMS approaches requires further validation (Jylänki et al., 2022). This evidentiary uncertainty risks undermining policymakers’ and practitioners’ confidence in the universal applicability of FMS interventions, potentially compromising their implementation fidelity in curricular design.

Early childhood represents a pivotal window for optimizing lifelong health trajectories, with the World Health Organization (WHO, 2023) advocating integrated approaches to support holistic development. However, a critical synthesis specifically targeting FMS interventions in typically developing preschoolers is lacking. While previous meta-analyses have examined the effects of physical activity on cognitive and social–emotional outcomes (Grady et al., 2025), they often encompass broad physical activity types or wider age ranges, diluting the specific insights into FMS mechanisms during the pivotal preschool period. Furthermore, existing reviews have not adequately addressed how intervention characteristics (e.g., “pure” motor skill training versus “combined” programs integrating cognitive or social elements) moderate the effects on EFs and SEC. This gap is significant because understanding these moderators is essential for designing targeted and effective interventions.

To address these limitations, the present meta-analysis is necessary for two primary reasons. First, it focuses exclusively on FMS interventions in typically developing preschoolers (aged 3–6 years), providing a precise estimate of their efficacy on dual developmental domains (EFs and SEC). Second, it systematically investigates key moderators—namely, intervention type (“pure” vs. “combined”), participant age, and intervention dosage—to elucidate the conditions under which FMS interventions are most effective. This research provides evidence-based practices for early childhood education and public health policy while advancing an integrated understanding of motor-cognitive-affective development in preschoolers.

## 2 Methods

This systematic review adheres to the PRISMA guidelines (Moher et al., 2009). The protocol is registered with PROSPERO (CRD420251073707).

### 2.1 Search strategy

Six electronic databases were subjected to comprehensive literature searches: PubMed, Web of Science, APA PsycINFO, ERIC, Scopus, and SPORT Discus. The search covered publications from January 1, 2000, to April 1, 2025, ensuring a 25-year scope to capture evolving trends and recent advancements in the field. Boolean search syntax was applied: (“fundamental motor skill*” OR “fundamental movement skill*” OR “basic motor skill*” OR gross motor* OR “motor competence” OR FMS) AND (intervention* OR program* OR training* OR “training method” OR “training approach”) AND (“executive function*” OR “inhibitory control” OR “working memory” OR “cognitive flexibil*”) AND (“social–emotional” OR “social behaviour” OR “emotional development”) AND (preschool* OR “early childhood”).

### 2.2 Eligibility criteria

The inclusion criteria were defined via the PICOS framework (McKenzie et al., 2019), with studies selected according to the following criteria: (1) participants were healthy preschool children (aged 3–6 years); (2) interventions comprised experimental or quasi experimental programs focused on FMS (e.g., running, jumping, throwing, kicking); (3) primary outcomes assessed EFs (inhibitory control, working memory, cognitive flexibility) and/or SEC (e.g., social behaviors, emotion regulation); (4) study design was a randomized controlled trial (RCT), non-randomized controlled trial, or single-group pretest–posttest design; (5) publication was in English; (6) full text was available and provided sufficient statistical data for meta-analysis (e.g., means, standard deviations, sample sizes).

Studies were excluded on the basis of the following criteria: (1) were commentaries, conference abstracts, notes, and summaries; (2) were individuals with diagnoses of disease, disorder, or obesity; (3) were interventions delivered in home or community settings; (4) were participants outside the age range of 3–6 years; (5) did not report any EFs or SEC outcomes or lacked detailed data; and (6) were duplicated publications or overlapping datasets.

### 2.3 Study selection

A rigorous procedure was followed in this systematic review. First, all retrieved records were imported into Endnote for duplication to ensure an independent and complete sample. Subsequently, two reviewers independently assessed the titles and abstracts of the retained records, with discrepancies resolved through consensus. Records meeting the eligibility criteria progressed to full-text review and data extraction; records not initially meeting the criteria also underwent full-text review to avoid excluding potentially eligible studies. Reviewer disagreements were resolved through consensus discussion, with third-party arbitration for persistent disagreements.

### 2.4 Quality assessment

Two independent reviewers assessed methodological quality via the Physiotherapy Evidence Database (PEDro) scale, which ranges from 0 to 10 points (Cashin and McAuley, 2020). This scale evaluates 11 criteria, with items 2–11 contributing to the total score. Higher scores indicate superior methodological quality. Two independent reviewers evaluated each study. The inter-rater agreement prior to consensus was calculated using Cohen’s kappa statistic, which indicated a substantial level of agreement (*κ* = 0.78). Discrepancies were resolved through discussion or adjudication by a third reviewer. On the basis of the developers’ recommendations (Cashin and McAuley, 2020), total scores were categorized as follows: < 4 points: Poor; 4–5 points: Fair; 6–8 points: Good; and 9–10 points: Excellent.

### 2.5 Data extraction

Dual independent data extraction was conducted for all eligible studies. The extracted information encompassed the following: study characteristics (including geographical location, design, and sample size), participant demographics (age and gender of children), outcome measures related to EFs and SEC, details of the intervention (type, content, frequency, duration), descriptions of the control conditions, and statistical data necessary for calculating effect sizes. The corresponding authors were contacted via email to clarify ambiguous or missing essential data.

### 2.6 Data synthesis and analysis

Meta-analytic computations were performed via Comprehensive Meta-Analysis (CMA) software (Borenstein, 2022). Studies were eligible for inclusion in the meta-analysis if they reported group sample sizes and post intervention means with corresponding measures of variability (standard deviation or standard error). In line with conventional procedures, post intervention values were used to compute standardized mean differences (SMDs) along with their 95% confidence intervals (CIs). A random-effects model was applied to explain between-study heterogeneity and to estimate the pooled effect size across studies. The magnitude of the effect size was interpreted on the basis of conventional thresholds: small (SMD = 0.2), medium (SMD = 0.5), and large (SMD = 0.8) (Cohen, 2013).

## 3 Investigation of heterogeneity and subgroup analyses

The *I*^2^ statistic was used to quantify heterogeneity among the included studies(Higgins and Thompson, 2002). Sources of heterogeneity were explored via subgroup and sensitivity analyses. On the basis of the integrated development of motor, cognitive, and affective domains and their age-dependent variations, three *a priori* hypotheses were formulated to investigate potential causes of heterogeneity: (1) Differential intervention effects arise from distinct types of interventions; (2) interventions targeting different age groups yield divergent outcomes; and (3) various intervention dosages result in differential effect magnitudes. To test these hypotheses, subgroup analyses were conducted according to intervention type, participant age, and intervention dosage. Exploratory analyses were warranted because of significant variation in intervention protocols across most included studies.

## 4 Results

### 4.1 Overview of studies

**Figure 1:**
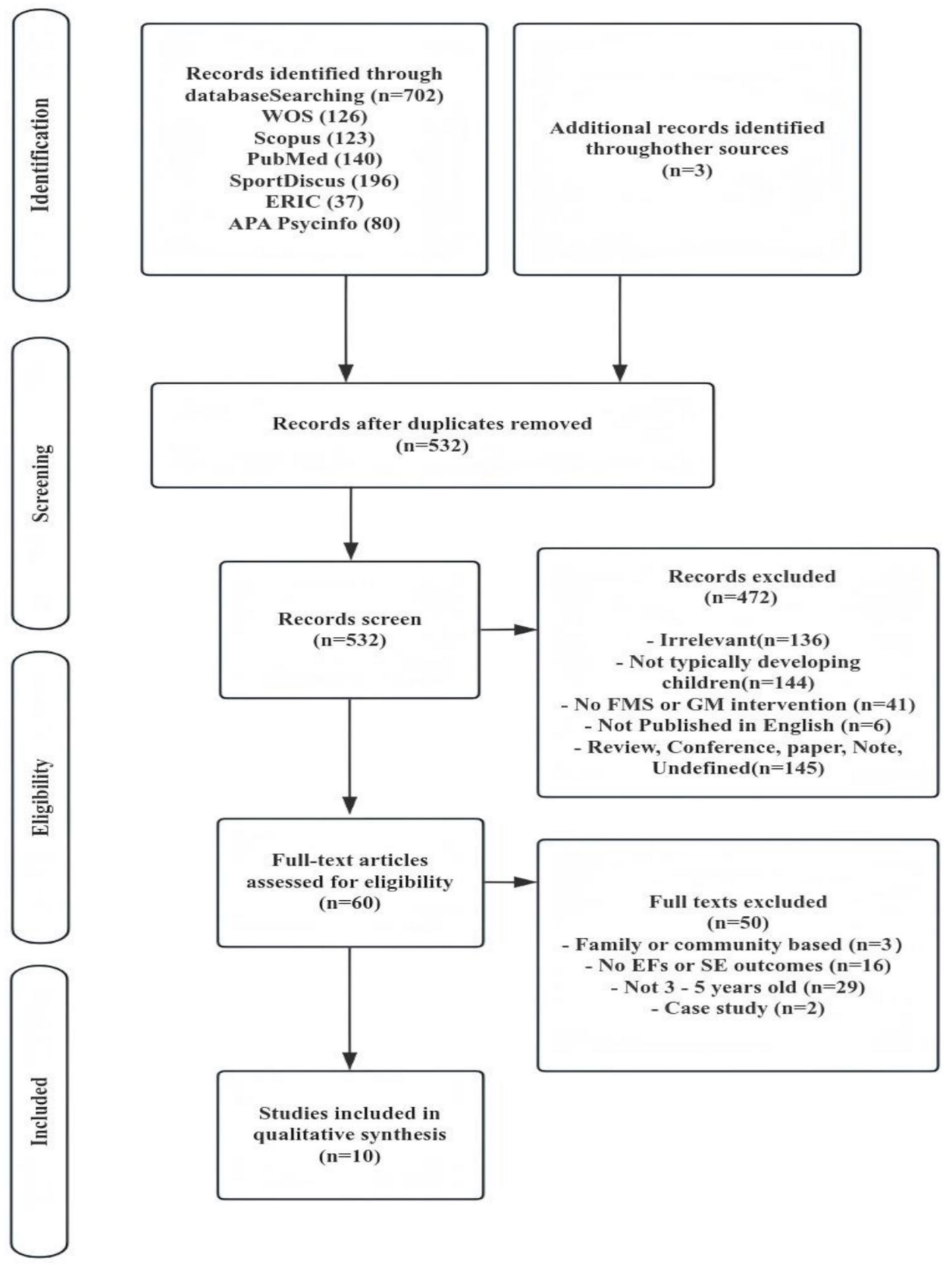
Article identified process (PRISMA).

The database searches identified 705 records, with three additional studies sourced elsewhere. After duplication (*n* = 182), 523 records underwent title and abstract screening. Next, 60 articles were subjected to full-text review. Finally, 10 studies met the inclusion criteria (Figure 1).

### 4.2 Study quality assessment

Dual independent PEDro assessments were conducted for the included studies (n = 10), with disagreements adjudicated by a third reviewer. On the basis of established cut-offs (Cashin and McAuley, 2020), six studies (60%) demonstrated good quality (scores of 6–8), whereas four (40%) were rated **Fair** (scores of 4–5). No studies were classified as excellent (9–10) or poor (<4).

The key methodological findings include high compliance in randomization (Part 2: 90%) and reporting of variability measures (Part 11: 100%). Critical limitations were observed: no study implemented allocation concealment (Part 3); therapist blinding was universally absent (Part 6); only 10% achieved participant blinding (Part 5); and intention-to-treat analysis was applied in only 20% of the studies (Part 9). These methodological gaps may introduce selection and performance biases, potentially leading to overestimation of intervention effects (see Table 1).

**Table 1:** PEDro scale compliance.

Study (Author, year)	Part 1	Part 2	Part 3	Part 4	Part 5	Part 6	Part 7	Part 8	Part 9	Part 10	Part 11	Score
Ortin et al. (2024)	✔	✔	–	✔	✔	–	✔	✔	–	✔	✔	7
Capio et al. (2024)	✔	✔	–	✔	–	–	–	✔	✔	✔	✔	6
Brian et al. (2024)	✔	✔	–	✔	–	✔	✔	–	–	✔	✔	6
Vazou and Mavilidi (2021)	–	✔	–	✔	–	–	✔	✔	–	✔	✔	6
Mulvey et al. (2018)	–	✔	–	✔	–	–	✔	✔	–	✔	✔	6
Cunningham et al. (2025)	✔	✔	–	✔	–	–	–	✔	–	✔	✔	5
Miller et al. (2023)	✔	✔	–	✔	–	–	–	✔	–	✔	✔	5
Hudson et al. (2021)	✔	✔	–	✔	–	–	–	✔	–	✔	✔	5
Piek et al. (2015)	✔	✔	–	–	–	–	–	✔	✔	✔	✔	5
Sendil et al. (2024)	✔	–	–	✔	–	–	–	✔	–	✔	✔	4

Part 1, eligibility criteria specified; Part 2, random allocation; Part 3, concealed allocation; Part 4, baseline group comparability; Part 5, participant blinding; Part 6, therapists blinding; Part 7, assessor blinding; Part 8, attrition rate  < 15%; Part 9, intention-to-treat analysis; Part 10, between-group statistical comparisons; Part 11, point estimates and variability data.

### 4.3 Participant characteristics

The included studies, published between 2015 (Piek et al., 2015) and 2025 (Cunningham et al., 2025), spanned six countries across four continents. North America: United States (Brian et al., 2024; Hudson et al., 2021; Miller et al., 2023; Mulvey et al., 2018; Vazou and Mavilidi, 2021); Europe: United Kingdom (Cunningham et al., 2025), Spain (Ortin et al., 2024); Asia: China (Capio et al., 2024), Turkey (Sendil et al., 2024); Oceania: Australia (Piek et al., 2015) (see Table 2).

**Table 2:** Intervention characteristics of the included studies.

Reference	Study design	Sample size int/con gender (female %)	Age (mean ± SD)	Intervention exposure	Pure or combine FMS intervention	Activities in control group	Measurement	Effect size (*d*)
Vazou and Mavilidi (2021), USA	Cluster RCT	138/135 Female 45.42%	4.22 ± 0.61 years	About 10 min, 5 times a week For 8 weeks	Combine. Move for Thought (M4T) program integrates fundamental gross motor skill development with whole-child pedagogy targeting physical, cognitive, social, and emotional domains.	Maintaining routine activities	EFs: Day/Night SEC: Social Skills Rating Scale questionnaire	EFs: 0.141 SEC: 0.080
Cunningham et al. (2025) , England	Cluster RCT	133/81 Female 47.20%	4.67±/N years	About 30 min, 1–2 times a week For 12 weeks	Combine. Movement and story-telling (MAST) program, ten FMS were covered, Vocabulary and comprehension were scaffolded through guided discussions of narrative illustrations.	Maintaining routine activities	EFs: Head-Toes-Knees Shoulders (HTKS) SEC: NA	EFs: 0.061 SEC: NA
Ortin et al. (2024), Spain	Cluster RCT	47/21 Female 50.00%	5.46 ± 0.22 years	About 30 min, 4 times a week For 3 weeks	Combine. ActivaMotricidad Program, it systematically unites fundamental motor skill development with cognitive challenges and social problem-solving demands.	Maintaining routine activities	EFs: Head-Toes-Knees Shoulders (HTKS) SEC: Interpersonal Problem-Solving Test (TREPI)	EFs: 0.904 SEC: 0.717
Piek et al. (2015), Australia	Cluster RCT	265/221 Female 49.71%	5.42 ± 0.30 years	About 30 min, 4 times a week For 10 weeks	Combine. Animal Fun program, it was designed to promote motor competence and social development through playful activities designed to boost children’s physical confidence.	Maintaining routine activities	EFs: NA SEC: Strengths and Difficulties Questionnaire (SDQ-T)	EFs: NA SEC: 0.063
Brian et al. (2024), USA	Cluster RCT	327/148 Female 46.32%	5.53 ± 0.68 years	About 10 min, twice a week For 36 weeks	Combine. Skipping With PAX, the intervention combines the PAX Good Behavior Game (a positive behavior support strategy) with the SKIP motor development curriculum, co-implemented during physical education sessions.	Maintaining routine activities	EFs: NA SEC: Social Skill Improvement System (SSIS)	EFs: NA SEC: 0.204
Miller et al. (2023), USA	Cluster RCT	67/45 Female 60.71%	4.45 ± 0.27 years	About 30 min, 3 times a week For 16 weeks	Pure. The Children’s Health Activity Motor Program (CHAMP) targets tripartite outcomes: motor skill proficiency, physical activity engagement, and perceived motor competence	Maintaining routine activities	EFs: Head-Toes-Knees Shoulders (HTKS) SEC: NA	EFs: 0.648 SEC: NA
Capio et al. (2024), China	Cluster RCT	95/90 Female 47.03%	3.96 ± 0.68 years	About 10 min, 3 times a week For 16 weeks	Pure. The intervention systematically targeted all three fundamental movement skill domains: (a) object control skill (throwing, kicking), (b) locomotor (running, hopping), and (c) stability skills (balancing, twisting).	Maintaining routine activities	EFs: Head-Toes-Knees Shoulders (HTKS) SEC: Social Competence and Behavior Evaluation Short Form (SCBE)	EFs: 0.492 SEC: 0.185
Hudson et al. (2021), USA	Cluster RCT	27/26 Female 58.49%	4.3 ± 0.6 years	About 20 min, twice a week For 8 weeks	Pure. The motor skills curriculum synergistically combined gross motor protocols from Young Athletes with fine motor activities from Finger Gym, creating a developmentally comprehensive intervention.	Maintaining routine activities	EFs: Executive Function Touch (EF Touch) SEC: NA	EFs: 0.587 SEC: NA
Sendil et al. (2024), Turkey	Quasi-experimental study	18/23 Female 34.15%	5.75 ± 0.23 years	About 30 min, twice a week For 8 weeks	Pure. The purpose-oriented basic movement patterns included open-ended tasks progressively advanced from simple to complex challenges.	Maintaining routine activities	EFs: Early Years Toolbox (EYT) SEC: NA	EFs: 0.035 SEC: NA
Mulvey et al. (2018), USA	Cluster RCT	50/57 Female 54.21%	5.14 ± 0.81 years	About 30 min, twice a week For 6 weeks	Pure. Successful Kinesthetic Instruction for Preschoolers (SKIP) program, it targets fundamental locomotor (e.g., run, jump) and object control skills (e.g., throw, kick).	Maintaining routine activities	EFs: Head-Toes-Knees Shoulders (HTKS) SEC: NA	EFs: 0.479 SEC: NA

The total sample comprised 2,039 participants (1,038 boys, 991 girls). The mean participant age ranged from 3.96 ± 0.68 years (Capio et al., 2024) to 5.75 ± 0.23 years (Cunningham et al., 2025), with age-specific clustering: 5-year-olds: 5 studies (Brian et al., 2024; Mulvey et al., 2018; Ortin et al., 2024; Piek et al., 2015; Sendil et al., 2024); 4-year-olds: 4 studies (Cunningham et al., 2025; Hudson et al., 2021; Miller et al., 2023; Vazou and Mavilidi, 2021); 3-year-olds: 1 study (Capio et al., 2024). All participants were generally healthy children; no study included individuals with health conditions or overweight/obesity.

### 4.4 Intervention characteristics

All included studies employed parallel-group designs, with 9 cluster randomized controlled trials (RCTs) and 1 quasi experimental design. Six studies were conducted in preschool/kindergarten environments; 3 in child development centers serving low-income families; and 1 in a reception class within a primary school. Five programs were implemented by research teams (experts in educational sciences, movement sciences, and rehabilitation sciences), 5 were delivered by trained preschool teachers. The control groups maintained standard educational routines (e.g., regular instruction, free play), with no specialized motor skill program.

The included studies featured five pure FMS interventions and five combined FMS programs. The intervention duration ranged substantially from 3 weeks (Ortin et al., 2024) to 36 weeks (Brian et al., 2024), with training sessions ranging from 1 time (Cunningham et al., 2025) to 5 times (Vazou and Mavilidi, 2021). Individual session lengths varied between 10 min (Ortin et al., 2024) and 45 min (Miller et al., 2023), although most of these sessions lasted 20–35 min. This dosage profile suggested that higher-frequency, shorter-duration interventions may enhance efficacy.

### 4.5 Outcome and measures

Intervention effects on EFs and SEC were systematically evaluated, with *a priori* subgroup analyses exploring heterogeneity through three specified moderators: (1) Intervention type (Pure FMS vs. Combined FMS), (2) Age cohort (3-year, 4-year, and 5-year subgroups), and (3) Intervention dose dichotomous as low-dose (≤2 sessions/week × 30 min/session) versus high-dose (>2 sessions/week × 30 min/session).

#### 4.5.1 Effects on executive functions

Eight studies reported EFs outcomes. Five studies demonstrated significant positive intervention effects, with three explicitly indicating that FMS interventions yielded significant improvements in *both* motor competence and EFs among preschoolers (Capio et al., 2024; Hudson et al., 2021; Mulvey et al., 2018). Three studies reported non-significant findings: two showed that FMS interventions significantly enhanced motor skills but not EF (Cunningham et al., 2025; Sendil et al., 2024); one combined-intervention study reported no significant between-group differences but identified significant main effects of time for EF development (Vazou and Mavilidi, 2021).

Meta-analysis revealed a significant small effect size for FMS interventions on EFs (*SMD* = 0.401, 95% CI: 0.195 to 0.607, *p* < 0.001) (see Supplementary Image 1). These results suggest that FMS interventions appear promising for the integrated development of preschoolers, albeit with inconsistent outcomes, further indicating that heterogeneous intervention effects are potentially moderated by program characteristics and individual factors.

##### 4.5.1.1 Subgroup analysis by intervention type

Five studies examined pure FMS interventions, and three evaluated combined FMS approaches. Analysis revealed that pure FMS interventions (*k* = 5) had moderate effects on EFs (*SMD* = 0.494, 95% CI: 0.315 to 0.674, *p* < 0.001). In contrast, combined FMS interventions (*k* = 3) had a smaller, non-significant effect (*SMD* = 0.317, 95% CI: −0.121 to 0.756, *p* = 0.156). This pattern suggests that pure FMS interventions may be more beneficial for enhancing EFs in preschool children. These findings contrary evidence from Jylänki et al. (2022), warranting further critical discussion.

##### 4.5.1.2 Subgroup analysis by age cohort

A combined younger cohort (3–4 years; *k* = 5) was formed for comparison against the 5-year-old cohort (*k* = 3). This pooling was justified based on two considerations: (1) developmental proximity, as 3- and 4-year-olds are both in the early preschool stage characterized by rapid foundational development of executive and motor skills, distinct from the more advanced cognitive profile of 5-year-olds who are nearing the transition to formal schooling; and (2) methodological necessity, to increase statistical power stability given the limited number of studies (*k* = 1) specifically targeting 3-year-olds. Analyses revealed significantly increased EFs in both groups, with 5-year-olds showing near-moderate effects (SMD = 0.492, 95% CI [0.061, 0.924], *p* = 0.025) and the 3–4-year cohort demonstrating smaller but robust effects (SMD = 0.359, 95% CI [0.115, 0.604], *p* = 0.004). This finding indicates greater but less stable efficacy potential in older preschoolers (evidenced by wider CI spans), whereas younger children presented more consistent and contextually generalize intervention benefits.

##### 4.5.1.3 Subgroup analysis by intervention dosage

Meta-analysis of four high-dosage studies (>2 sessions/week × 30 min/session; total >60 min/week) demonstrated moderate, statistically significant improvements in EFs (*SMD* = 0.513, 95% CI [0.237, 0.790], *p* < 0.001), whereas four low-dosage studies (≤2 sessions/week × min/session; ≤60 min/week) showed small, marginally significant effects (*SMD* = 0.270, 95% CI [−0.007, 0.547], *p* = 0.056). This dose–response pattern establishes exceeding the 60-min weekly threshold as a critical determinant for robust cognitive benefits, confirming that an adequate intervention dosage is essential for optimizing FMS efficacy in preschoolers.

#### 4.5.2 Effects on social–emotional competence

Five studies assessed SEC. Two studies demonstrated significant benefits, with Brian et al. (2024) reporting concurrent improvements in motor and social skills, whereas Ortin et al. (2024) advocated combined FMS interventions as cost-effective tools for holistic development. Conversely, three studies showed non-significant overall effects, with Capio et al. (2024) and Vazou and Mavilidi (2021) finding no domain-specific improvements, although Piek et al. (2015) observed selective gains in prosocial behaviors despite null primary outcomes.

Random effects meta-analysis revealed a significant but weak overall effect of FMS interventions on SEC (*SMD* = 0.163, 95% CI [0.028, 0.299], *p* = 0.018) (see Supplementary Image 2). These results indicate that while FMS interventions produce statistically detectable benefits for social–emotional competence, their clinical significance may be limited. Substantial heterogeneity (*I*^2^ = 68%) suggests that treatment effects are likely moderated by program characteristics (e.g., intervention type, dosage) and individual factors (e.g., baseline development, socioeconomic status).

##### 4.5.2.1 Subgroup analysis by intervention type

Four combined FMS interventions revealed a minimal effect size approaching statistical significance (*SMD* = 0.169, 95% CI [−0.002, 0.340], *p* = 0.052), whereas the single pure FMS study (*k* = 1) showed a comparable point estimate (*SMD* = 0.185, 95% CI [−0.103, 0.474], *p* = 0.208) but was excluded from pooled analysis because the minimum study threshold (*k* ≥ 2) for reliable interpretation was violated. These patterns tentatively suggest that combined approaches may be more conducive to SEC, although statistical limitations preclude definitive conclusions.

##### 4.5.2.2 Subgroup analysis by age

Three studies with 5-year-olds revealed marginally significant small effects on SEC (*SMD* = 0.223, 95% CI [−0.022, 0.467], *p* = 0.074), whereas two studies with 3–4-year-olds showed non-significant minimal effects (*SMD* = 0.122, 95% CI [−0.061, 0.306], *p* = 0.191). This differential efficacy profile suggests that the 5-year developmental window may represent a more responsive period for social–emotional intervention than younger preschool ages do.

##### 4.5.2.3 Subgroup analysis by intervention dosage

Five studies employed high-dosage interventions (>2 sessions/week × 30 min/session; total >60 min/week), whereas only one utilized a low dosage (≤2 sessions/week × 30 min/session; ≤60 min/week). A meta-analysis of high-dosage studies revealed a non-significant minimal effect on SEC (*SMD* = 0.163, 95% CI [−0.022, 0.349], *p* = 0.084). Low-dosage studies (*k* = 1) were excluded from pooled analysis because the minimum study threshold (*k* ≥ 2) for reliable interpretation was violated. These results suggest that higher-dose FMS interventions may not sufficiently enhance social–emotional development, whereas the near absence of low-dosage studies indicates a critical evidence gap and potential selection bias in current research paradigms.

### 4.6 Heterogeneity analysis

#### 4.6.1 Executive functions

Subgroup analyses revealed moderate heterogeneity in EFs (*I*^2^ = 52.4%, *p* = 0.040). Significant between-subgroup differences were observed for intervention type (*p* = 0.041), with combined FMS interventions exhibiting substantial heterogeneity (*I*^2^ = 73.9%, *p* = 0.022; *n* = 3), whereas pure FMS interventions showed negligible heterogeneity (*I*^2^ = 0.0%, *p* = 0.582; *n* = 5). No significant subgroup differences emerged for age (*p* = 0.292) or dose (*p* = 0.060). These findings indicate that intervention type emerged as a potential moderator explaining differences in EF outcomes, a pattern predominantly driven by design variations in combined FMS programs, whereas age and dosage are not significant moderators.

Sensitivity analysis demonstrated that excluding Ortin et al. (2024) from combined FMS interventions reduced the pooled effect size to *SMD* = 0.088 (95% CI: −0.136, 0.312), indicating high sensitivity to this single study. Conversely, age and dose subgroups maintained statistical robustness (*p* < 0.05) when any individual study was removed, confirming the stability of the primary findings.

#### 4.6.2 Social–emotional competence

Subgroup analyses indicated low heterogeneity in social–emotional outcomes overall (*I*^2^ = 33.5%, *p* = 0.018), with no significant between-group differences across intervention types, age cohorts, or dosage levels (*p* > 0.05), suggesting comparable treatment effects. However, substantial heterogeneity was observed within the combined FMS interventions (*I*^2^ = 62.9%, *p* = 0.068) and the 5-year-old cohort (*I*^2^ = 64.2%, *p* = 0.061), indicating that potential unexplored moderators require further investigation.

Sensitivity analysis demonstrated that excluding Ortin et al. (2024) from combined FMS interventions reduced the pooled effect size to *SMD* = 0.069 (95% CI: −0.073, 0.212). Exclusion of Vazou and Mavilidi (2021)or Piek et al. (2015) yielded increased point estimates but with substantial widening of confidence intervals. All the scenarios resulted in non-significant effects (*p* > 0.05), confirming the inconsistent efficacy of the combined FMS programs. Conversely, the original findings for both the 5-year-old cohort and the high-dose subgroup (>60 min/week) demonstrated robustness across all sensitivity analyses.

### 4.7 Publication bias analysis

Comprehensive assessment via funnel plots, Duval and Tweedie’s trim-and-fill, Egger’s regression, and classic fail-safe N revealed no significant publication bias (See Supplementary Images 3, 4). Funnel plots demonstrated essential symmetry, trim-and-fill detected zero missing studies, and Egger’s test revealed non-significant intercepts (EFs: *t* = 0.915, *p* = 0.395; SEC: *t* = 2.534, *p* = 0.085). While fail-safe *N* exceeded the 5 *k* + 10 threshold for EFs (*k* = 8, *N*_fs_ = 57), it fell below the threshold for the SEC (*k* = 5, *N*_fs_ = 9). This discrepancy reflects instability from limited SEC studies rather than methodological bias, thus not materially affecting evidence quality (Guyatt et al., 2008). Collectively, these analyses indicate robust protection against publication bias.

## 5 Discussion

The synchronous, holistic, and symbiotic development of motor, cognitive and affective development is theoretically grounded in dynamic systems theory (Thelen and Smith, 1994) and embodied cognition frameworks (Glenberg, 2010). This dynamic interdependence among domains is particularly salient during the preschool period (ages 3–6), which represents a sensitive transitional stage for FMS development. During this window, rapid advancements in motor development are inextricably linked to and facilitate parallel growth in cognitive control and emotional regulation. Fundamental motor skill (FMS) proficiency represents a critical developmental milestone in early childhood, with empirical evidence supporting its role in enhancing EFs and SEC (Mulvey et al., 2018; Ortin et al., 2024), although methodological inconsistencies persist (Capio et al., 2024; Sendil et al., 2024). Key knowledge gaps remain regarding optimal intervention types, age implementation windows, and dose–response thresholds. This ambiguity risks undermining policymakers’ and practitioners’ confidence in the universal applicability of FMS interventions, potentially impeding curriculum integration. While current reviews synthesize pedagogical approaches for preschoolers (Grady et al., 2025; Liu and Ying, 2021), these have not fully demonstrated the transformative educational value of FMS training during this developmental period. Consequently, current evidence remains insufficient to stimulate systemic policy reform or widespread teaching innovation.

This meta-analysis quantified the efficacy of fundamental movement skill (FMS) interventions for enhancing EFs and SEC in typically developing preschoolers, revealing a significant moderate effect on EFs (*SMD* = 0.401, 95% CI [0.252, 0.550], *p* < 0.001) that aligns with dynamic systems theory’s perception–action coupling mechanism (Thelen and Smith, 1994) and a modest yet significant SEC improvement (*SMD* = 0.163, 95% CI [0.028, 0.299], *p* = 0.018) that underscores the necessity for socially embodied contexts (e.g., cooperative play, emotional dialogue) to optimize intervention efficacy as per embodied cognition frameworks (Glenberg, 2010). The robustness of these findings is strengthened by the consistent pre-intervention training provided to instructors across studies, which enhanced implementation fidelity.

Subgroup analyses revealed moderate heterogeneity in executive function effects (*I*^2^ ≈ 52%) and low-to-moderate heterogeneity in social–emotional outcomes (*I*^2^ ≈ 33%), with the most substantial subgroup differences observed for intervention type. Compared with combined approaches, pure FMS interventions demonstrated significantly greater EF benefits (*SMD* = 0.494) (*SMD* = 0.311; between-group *p* = 0.041), suggesting that task-focused motor training better supports preschoolers’ executive development—although this finding contradicts previous findings (Jylänki et al., 2022). Potential mechanisms include the following: (1) Pure FMS protocols optimize sustained aerobic activity that may increase gray matter volume in prefrontal-caudate-hippocampal circuits (Erickson et al., 2015), and (2) combined programs often violate multiple resource theory (Wickens, 2008), inducing central attentional bottlenecks that impair cognitive-motor integration (Moreira et al., 2021; Pashler, 1994). Conversely, combined FMS approaches showed marginally significant SEC advantages (*SMD* = 0.169, *p* = 0.052), likely through naturalistic social embedding (Ortin et al., 2024) and targeted emotion-regulation activities (Piek et al., 2015), whereas pure FMS may limit affective learning owing to a narrow task focus. However, this finding should be interpreted with caution. The observed advantage is not robust, as indicated by the overlapping confidence intervals and the loss of statistical significance upon removal of a single study from the combined FMS group, which likely stem from the limited number of studies in the analysis. Therefore, while the data suggest a potential superiority of pure FMS interventions for targeting EFs, this conclusion should be considered preliminary and requires verification in future research with a larger and more balanced sample of studies.

Although age did not emerge as a primary moderator, subgroup analyses revealed critical developmental windows, with 5–6-year-olds demonstrating significantly greater intervention benefits for both EFs (*SMD* = 0.492) and SECs (*SMD* = 0.223) than 3–4-year-olds did (EF: *SMD* = 0.359; SEC: *SMD* = 0.122). This advantage likely stems from (1) accelerated prefrontal cortex (PFC) myelination enhancing neural signaling efficiency during the 5-year-old critical period (Diamond, 2013), whereas 3–4-year-olds remain in early PFC maturation stages, and (2) qualitative leaps in sociocognitive capacities enabling deeper social interactions characteristic of 5-year-olds (Feldman and Eidelman, 2009). Collectively, these neurodevelopmental and behavioral shifts identify age 5 as a potential golden window for movement-based interventions targeting EF and SEC development, warranting high-frequency protocols specifically optimized for this transitional stage.

Dosage analysis revealed significantly greater executive function benefits from high-dose interventions (>60 min/week; *SMD* = 0.513) than from low-dose interventions (≤60 min/week; *SMD* = 0.270), which aligns with evidence that EF gains require sustained practice exceeding minimum stimulation thresholds (Diamond and Ling, 2016; Simons et al., 2016). This finding supports findings that higher frequency/longer duration interventions optimize cognitive outcomes (Bockmann and Yu, 2023), as weekly frequencies ≤2 sessions appear insufficient for significant EF improvements (Cunningham et al., 2025). In contrast, no statistically significant dose–response advantage was found for social–emotional competence (SEC) development (>60 min/week: SMD = 0.163; ≤60 min/week: SMD = 0.204). Given the limited number of studies, this absence of a clear dose–response relationship cannot be conclusively interpreted as evidence for the true absence of an effect. Therefore, future research with a wider range of well-defined dosages is needed to fully explore this relationship. Collectively, these patterns indicate that EF development favors moderate-to-high intensity/frequency regimens, whereas SEC benefits may depend more on contextual richness than on dosage intensity.

Despite the multinational consensus of FMS as core preschool content (Council of Ministers of Education Canada, 2014; Ministry of Education China, 2012b; UK Department for Education, 2023), evidence-based pedagogical frameworks remain underdeveloped. To bridge this gap, we propose the following actionable recommendations for implementation: First, integration should be led by trained educators who can provide high-quality, explicit instruction in FMS within playful yet planned contexts. Second, programs should be designed to align motor learning goals with cognitive and socio-emotional objectives, for instance, by embedding FMS practice into storytelling sessions that also require following rules (cognitive) and cooperative games that encourage sharing and turn-taking (social–emotional). Third, scheduling should aim for brief, frequent sessions (e.g., 20–30 min, ≥3 times per week) integrated into the daily routine to meet the identified high-dosage threshold for cognitive benefits.

Collectively, these findings underscore the value of structured FMS interventions as a powerful, evidence-based strategy to promote integrated development in early childhood. Our results provide strong empirical support for the Nurturing care framework (World Health Organization et al., 2018). This echoes the WHO’s vision of nurturing care and integrated approaches to support the whole child. By demonstrating significant benefits in both executive functions and social–emotional competence, this meta-analysis justifies the institutionalization of FMS as foundational components of preschool curricula worldwide. Translating these findings into policy and practice will be crucial for advancing global early childhood development goals.

### 5.1 Limitations

This systematic review is limited by its exclusive focus on typically developing children, thereby excluding populations with special needs and potentially restricting the generalization of findings. Furthermore, the English-language publication requirement may have led to the omission of relevant non-English evidence. To mitigate these constraints, future research should prioritize more comprehensive investigations in these underrepresented domains to generate robust evidence with broader applicability.

## 6 Conclusion

This meta-analysis demonstrated that FMS interventions have significant benefits for preschool children’s EFs and SEC. Pure FMS interventions had stronger effects on EFs, whereas combined FMS interventions had stronger effects on the SEC, particularly when conducted at least twice a week and for 30 min each. These differential effects can be understood through the lens of embodied cognition and dynamic systems theories. The superior effect of pure FMS on EFs may result from a focused engagement of cognitive resources during repetitive skill practice, which efficiently strengthens neural pathways supporting cognitive control. In contrast, combined FMS interventions, by embedding motor activities within rich social and narrative contexts, directly provide opportunities for practicing emotional co-regulation and social interaction, thereby fostering SEC through embodied social experiences. These findings support the embodied and dynamic interplay between motor, cognitive, and affective development in early childhood. As such, FMS training represents a promising, developmentally appropriate strategy to promote comprehensive development. In line with the WHO’s call for integrated early childhood interventions, we recommend incorporating structured FMS programs into preschool curricula to maximize EFs and SEC for the integrated development of preschool children worldwide. Future studies should pursue four strategic directions: population expansion (special needs), mechanistic investigation (neurobiological, age-specific), methodological refinement (dosage, longitudinal tracking), and practical translation (cultural adaptation), to construct a more comprehensive evidence base.

## Data Availability

The datasets presented in this study can be found in online repositories. The names of the repository/repositories and accession number(s) can be found in the article/Supplementary material.
