# Supplementary figures and images for: Fundamental motor skill interventions significantly improve executive functions and social–emotional competence in preschoolers: a meta-analysis
Source: Front Psychol. 2026 Jan 9;16:1721589. doi: 10.3389/fpsyg.2025.1721589 (PMC12827644; doi:10.3389/fpsyg.2025.1721589)

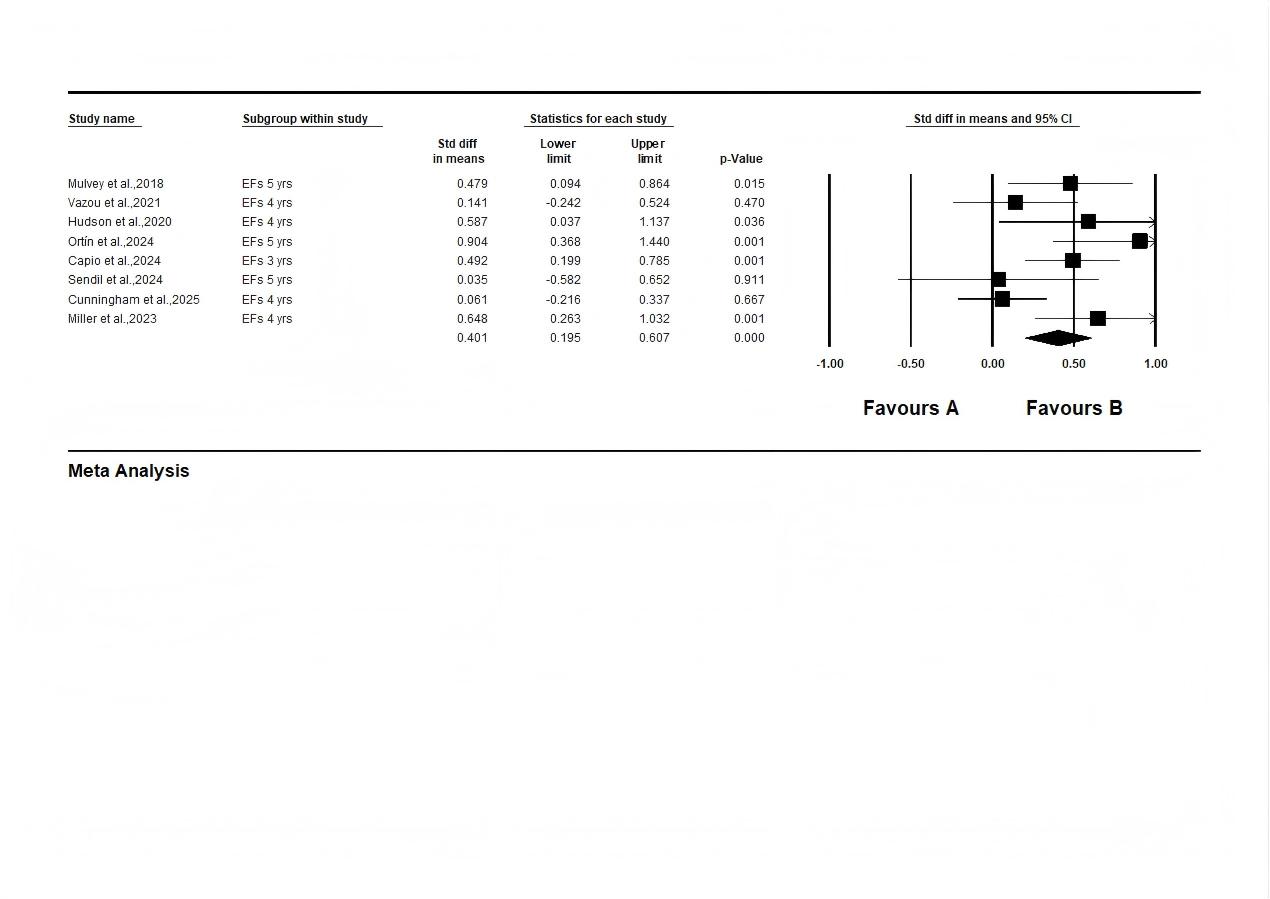

Supplement: Supplementary file 1 [file Image_1.jpeg]

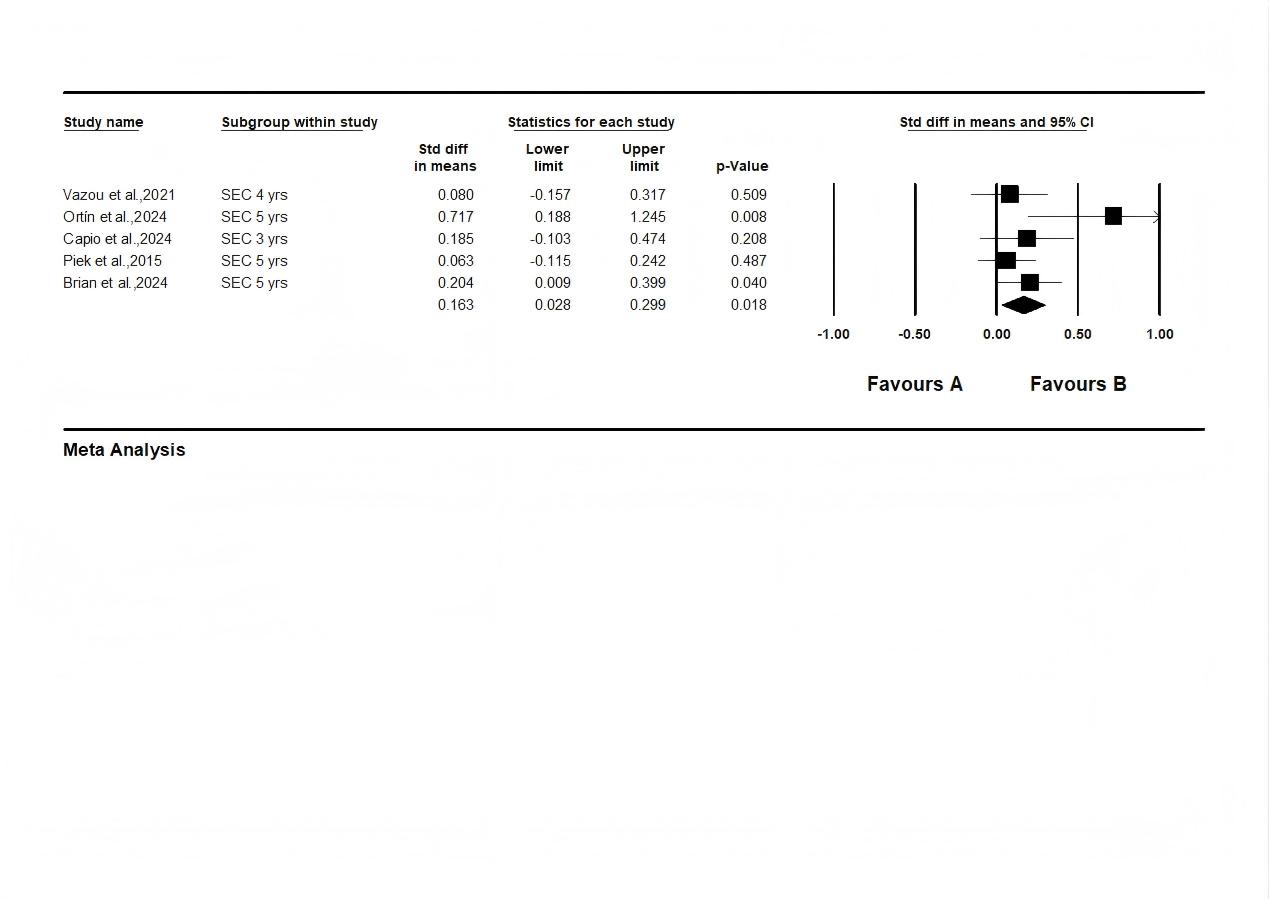

Supplement: Supplementary file 2 [file Image_2.jpeg]

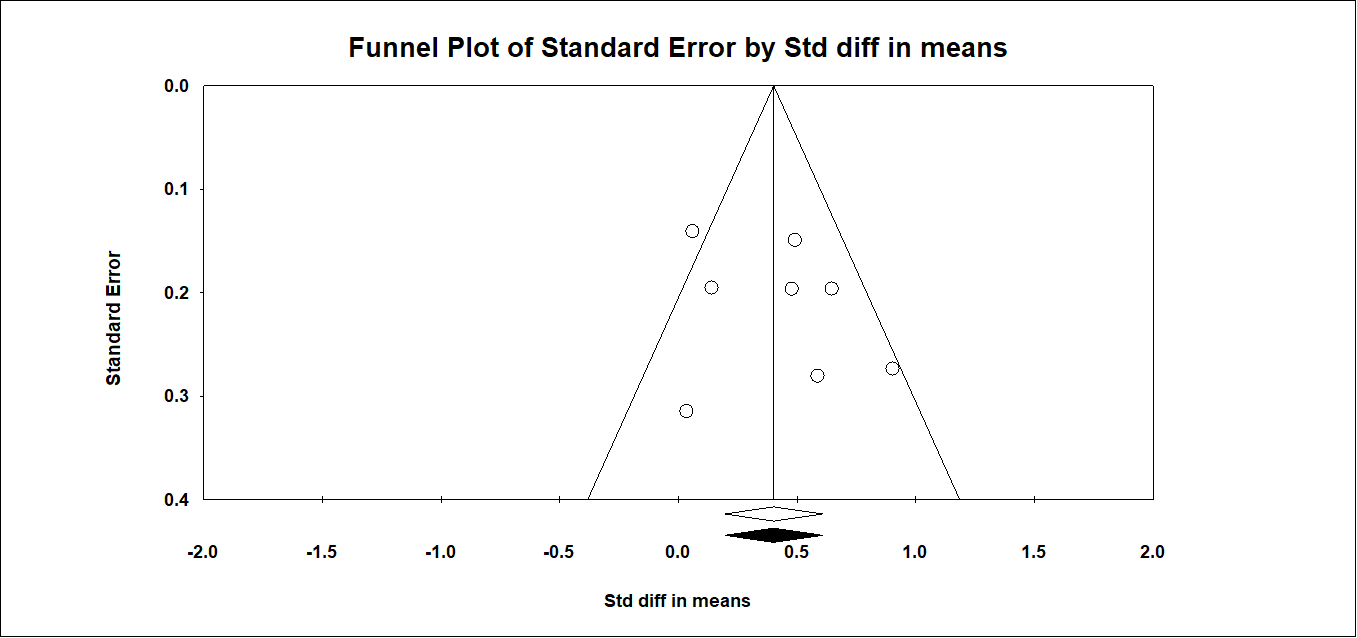

Supplement: Supplementary file 3 [file Image_3.png]

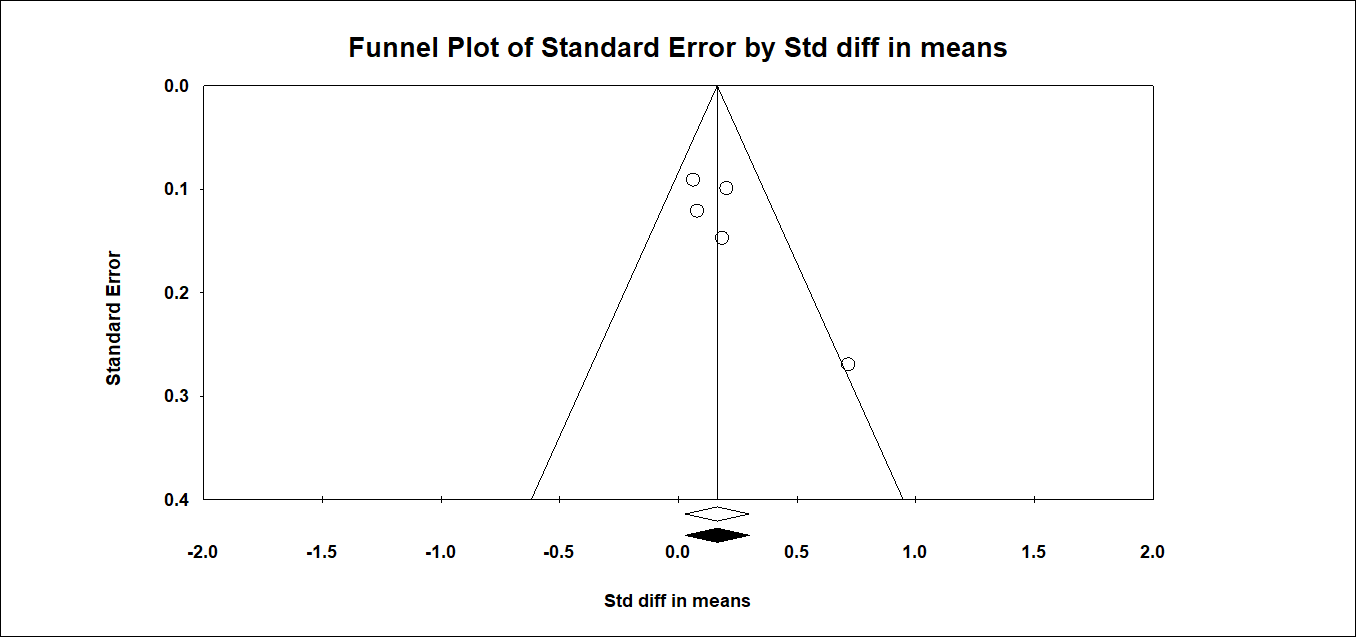

Supplement: Supplementary file 4 [file Image_4.png]
