# Supplementary material for: Fundamental motor skill interventions significantly improve executive functions and social–emotional competence in preschoolers: a meta-analysis
Source: Front Psychol. 2026 Jan 9;16:1721589. doi: 10.3389/fpsyg.2025.1721589 (PMC12827644; doi:10.3389/fpsyg.2025.1721589)
Supplement: Supplementary file 5 [file Table_1.DOCX]

**Supplementary Table S1. Intervention characteristics of the included studies**

| Reference | Study design | Sample size int/con  Gender(female %) | Age  ( mean ± SD) | Intervention exposure | Pure or Combine  FMS intervention | Activities in  control group | Measurement | Effect size  (d) |
| --- | --- | --- | --- | --- | --- | --- | --- | --- |
| Vazou et al.  (2021)  USA | Cluster RCT | 138/135  Female 45.42% | 4.22±0.61 yrs | About 10 minutes,  5 times a week  For 8 weeks | Combine. The Move for Thought (M4T) preK-K program integrates fundamental gross motor skill development with whole-child pedagogy targeting physical, cognitive, social, and emotional domains. | Maintaining routine activities | EFs:Day/Night  SEC:Social Skills Rating Scale questionnaire | EFs: 0.141  SEC: 0.080 |
| Cunningham et al. (2025)  England | Cluster RCT | 133/81  Female 47.20% | 4.67±/N  yrs | About 30 minutes,  1-2 times a week  For 12 weeks | Combine. Movement and story-telling (MAST) program, ten FMS were covered, Vocabulary and comprehension were scaffolded through guided discussions of narrative illustrations. | Maintaining routine activities | EFs:Head-Toes-Knees Shoulders (HTKS)  SEC: NA | EFs: 0.061  SEC: NA |
| Ortín et al.  (2024)  Spain | Cluster RCT | 47/21  Female 50.00% | 5.46±0.22  yrs | About 30 minutes,  4 times a week  For 3 weeks | Combine. ActivaMotricidad Program, it systematically unites fundamental motor skill development with cognitive challenges and social problem-solving demands. | Maintaining routine activities | EFs:Head-Toes-Knees Shoulders (HTKS)  SEC: Interpersonal Problem-Solving Test (TREPI) | EFs: 0.904  SEC: 0.717 |
| Piek et al.  (2015)  Australia | Cluster RCT | 265/221  Female 49.71% | 5.42 ± 0.30 yrs | About 30 minutes,  4 times a week  For 10 weeks | Combine. Animal Fun program, it was designed to promote motor competence and social development through playful activities designed to boost children's physical confidence. | Maintaining routine activities | EFs:NA  SEC:Strengths and Difficulties Questionnaire (SDQ-T) | EFs: NA  SEC: 0.063 |
| Brian et al.  (2024)  USA | Cluster RCT | 327/148  Female 46.32% | 5.53±0.68  yrs | About 10 minutes,  twice a week  For 36 weeks | Combine. Skipping With PAX, the intervention combines the PAX Good Behavior Game (a positive behavior support strategy) with the SKIP motor development curriculum, co-implemented during physical education sessions. | Maintaining routine activities | EFs:NA  SEC: Social Skill Improvement System (SSIS) | EFs: NA  SEC: 0.204 |
| Miller et al.  (2022)  USA | Cluster RCT | 67/45  Female 60.71% | 4.45±0.27  yrs | About 30 minutes,  3 times a week  For 16 weeks | Pure. The Children's Health Activity Motor Program (CHAMP) targets tripartite outcomes: motor skill proficiency, physical activity engagement, and perceived motor competence | Maintaining routine activities | EFs:Head-Toes-Knees Shoulders (HTKS)  SEC:NA | EFs: 0.648  SEC: NA |
| Capio et al.  (2024)  China | Cluster RCT | 95/90  Female 47.03% | 3.96±0.68  yrs | About 10 minutes,  3 times a week  For 16 weeks | Pure. The intervention systematically targeted all three fundamental movement skill domains: (a) object control (throwing, catching, rolling), (b) locomotor (running, jumping, hopping), and (c) stability skills (balancing, stretching, twisting). | Maintaining routine activities | EFs:Head-Toes-Knees Shoulders (HTKS)  SEC:Social Competence and Behavior Evaluation Short Form (SCBE） | EFs: 0.492  SEC: 0.185 |
| Hudson et al. (2020)  USA | Cluster RCT | 27/26  Female 58.49% | 4.3 ± 0.6  yrs | About 20 minutes,  twice a week  For 8 weeks | Pure. The motor skills curriculum synergistically combined gross motor protocols from Young Athletes with fine motor activities from Finger Gym, creating a developmentally comprehensive intervention. | Maintaining routine activities | EFs:Executive Function Touch （EF Touch)  SEC:NA | EFs: 0.587  SEC: NA |
| Sendil et al.  (2024)  Turkey | Quasi-experimental study | 18/23  Female 34.15% | 5.75 ± 0.23 yrs | About 30 minutes,  twice a week  For 8 weeks | Pure. The purpose-oriented basic movement patterns  included open-ended tasks progressively advanced from simple to complex challenges. | Maintaining routine activities | EFs:Early Years Toolbox(EYT)  SEC:NA | EFs: 0.035  SEC: NA |
| Mulvey et al. (2018)  USA | Cluster RCT | 50/57  Female 54.21% | 5.14 ± 0.81 yrs | About 30 minutes,  twice a week  For 6 weeks | Pure. Successful Kinesthetic Instruction for Preschoolers (SKIP) program, it targets fundamental locomotor (e.g., run, jump, hop) and object control skills (e.g., throw, catch, kick). | Maintaining routine activities | EFs:Head-Toes-Knees Shoulders(HTKS)  SEC:NA | EFs: 0.479  SEC: NA |
